# Psychogenic hearing loss in children and adolescents. Diagnosis and psychotherapy

**DOI:** 10.1080/07853890.2024.2447412

**Published:** 2025-01-15

**Authors:** Grażyna Gwizda, Anna Horaczyńska-Wojtaś, Grażyna Mielnik-Niedzielska, Anna Kasprzyk, Lechosław Paweł Chmielik, Artur Niedzielski

**Affiliations:** a Department of Pediatric Otolaryngology, Phoniatrics and Audiology, Medical University of Lublin, Lublin, Poland; b Department of Pediatric Otolaryngology, Centre of Postgraduate Medical Education, Warsaw, Poland

**Keywords:** Psychogenic hearing loss, hearing loss, children, psychogenic disorders, audiology

## Abstract

**Introduction:**

Psychogenic hearing loss is often neglected in the differential diagnosis of hearing disorders. In a difficult diagnostic process and treatment of psychogenic hearing loss disorder, the close cooperation of the audiologist, psychologist, patient, and his family is required. The study aimed to improve the knowledge and understanding of psychogenic hearing loss, establish a differential diagnosis in audiological tests in children, determine diagnostic procedures and finally apply adequate therapeutic procedures. The professional knowledge presented in the study will enable adaptation of reliable information which can be transferred to young patients and their families to let them recognize their problems.

**Material and methods:**

A group of 321 children, admitted to the Department of Pediatric Otorhinolaryngology, Phoniatrics and Audiology, The Children’s Hospital in Lublin, Poland, from January 2019 to December 2021, who complained of hearing loss were analysed in the study.

**Results:**

Twenty-two patients (15 girls and 7 boys) met the audiological criteria of psychogenic hearing loss and were enrolled to the study. Seventeen children complained of bilateral hearing loss, and five patients presented with a unilateral hearing loss.

**Conclusions:**

Family and personality problems cause unresolved difficulties in children and can lead to psychogenic deafness. Constant interest demonstrated by a guardian not only when the child is causing troubles or is complaining of disease is the key to avoiding developing psychosomatic disorders such as psychogenic hearing loss.

## KEY MESSAGES

1. Psychogenic hearing loss is often neglected in the differential diagnosis of hearing disorders.
2. Family and personality problems cause unresolved difficulties in children and can lead to psychogenic deafness.

## Introduction

Around 9% of paediatric population of Poland manifest mental health problems and requires professional help [1]. Some of the mental issues manifest in psychogenic disorders such as psychogenic hearing loss (PHL) which can be also known as pseudohypoacusis, functional or non-organic hearing loss. PHL is defined as hearing loss that cannot be explained by anatomic or physiologic abnormalities. According to the literature, the psychogenic hearing loss represents 1-3% of all cases of hearing loss disorders diagnosed in childhood [2,3]. It affects around 7% of children aged 6 to 17 years old [4]. This number suggests a few cases per month in typical clinical practice [5]. The factual incidence of PHL may be largely underestimated due to similarity of symptoms to different hearing disorders. Although there have been many studies on hearing loss in children, little is known about causes and pathogenesis of PHL.

The manifestation of psychosomatic disorders involving hearing in audiological tests is often questioned and interests contemporary audiological practitioners. The results of objective hearing tests exhibit deficits that cannot be explained by organic pathology and were documented in research papers by Doerfler and Stewart (1946), Chaiklin and Ventry (1963), Noble (1987) and Yamamoto et al. (1991) and others [6].

Due to advances in objective audiological procedures, the diagnosis of PHL is less challenging, while proper management and adequate therapeutic procedures remain problematic. According to the American Psychiatric Association, PHL is categorized as conversion disorder and is differentiated from malingering and factitious disorders. The conversion type exhibits suppressed emotions, leading unaware patients to give a disproportional hearing threshold in objective and subjecting hearing tests [6,7].

The study aimed to improve the knowledge and understanding of psychogenic hearing loss, establish a differential diagnosis in audiological tests in children, determine diagnostic procedures and finally apply adequate therapeutic procedures. It aimed to identify typical psychogenic hearing loss findings on audiological tests to minimize diagnostic measures and the duration of diagnosis. The professional knowledge presented in the study will enable adaptation of reliable information which can be transferred to young patients and their families to let them recognize their problems [8]. The authors would also like to emphasize how important a patient’s well-being is for an appropriate audiological procedure that allows making a proper diagnosis [7].

## Materials and methods

In the present study, we conducted a retrospective analysis for 321 patients who complained of impaired hearing and were admitted with the initial diagnosis of hearing loss to the Department of Pediatric Otorhinolaryngology, Phoniatrics and Audiology, The Children’s Hospital in Lublin, Poland from January 2019 to December 2021. Among this group, 22 patients met the audiological criteria of PHL, which is discrepancy between subjective (pure tone audiometry) and objective audiometry (otoacoustic emissions, auditory brain response examination).

The following clinical data sets were analysed: history and psychological evaluation, an ENT (ear, nose, throat) examination, audiometric tests (pure tone audiometry, PTA, impedance audiometry, IA, otoacoustic emission, OAE, auditory brain response examination, ABR). The following grades of hearing impairment were used for classification of hearing loss according to the initial PTA results (average of 500, 1000, 2000 and 4000 Hz):

- Mild hearing loss: 26–40 dB,
- Moderate hearing loss: 41–55 dB,
- Moderately severe hearing loss: 56–70 dB,
- Severe hearing loss: 71–90 dB,
- Profound hearing loss: 91 dB or greater.

In the psychological examination, the intellectual development Wechsler D intelligence scale for children was applied [9] and speech development was assessed. Adaptation skills were evaluated by a sentence completion test [10]. Patients underwent psychological interviews and examinations of the family situation using a genogram technique [11]. First, the examination aimed to exclude the anatomical background of hearing loss and next to confirm the psychogenic background of hearing loss. The final phase was recognizing the disorder’s psychological context and focusing on therapeutic actions and psychotherapy.

The study was conducted according to the guidelines of the Declaration of Helsinki and approved by the Bioethics Committee of the Medical University of Lublin - KE-0254/7/2013. Informed and written consent was obtained from the parents/legal guardians of the participants to participate in this study. They were informed of the possibility of discontinuing participation in the study at any time during the study.

## Results

Twenty-two children (6,85% of patients administered to the hospital with suspected hearing loss) were diagnosed with PHL. Among our study group there were 15 girls and 7 boys (ratio 2,14:1). The mean age of the patient group was 12,41 years (range: 7–16). 17 patients (77,27%) complained of a bilateral hearing loss, which was mostly symmetric. Five patients presented with an unilateral hearing impairment.

The results of PTA showed two patients with moderate hearing loss, seven patients with moderately severe hearing loss, eight patients with severe hearing loss and five patients with profound hearing loss. Mean air conduction threshold was 66,32 dB on left ears and 75,27 dB on right ears, whereas bone conduction threshold was 61,52 dB on left ears and 68,32 dB on right ears. Detailed results are presented in Table 1.

**Table 1.** Clinical characteristic of patients diagnosed with psychogenic hearing loss.

Case No.	Lesion site	Hearing loss grade	Hearing loss dB HL		Type of audiogram
			Left ear	Right ear	
1	Bilateral	Moderately severe, 66 dB	65	67	Flat
2	Bilateral	Severe, 73 dB	76	70	Flat
3	Right	Moderately severe, 56 dB	11	56	Flat
4	Bilateral	Moderate, 47 dB	44	51	Flat
5	Bilateral	Severe, 84 dB	81	87	Profound
6	Bilateral	Moderately severe, 68 dB	74	62	Descending
7	Left	Severe, 82 dB	82	10	Profound
8	Bilateral	Severe, 73 dB	71	76	Flat
9	Bilateral	Moderately severe, 67 dB	69	66	U-shape
10	Bilateral	Severe, 78 dB	75	81	Descending
11	Bilateral	Moderately severe, 56 dB	55	57	Flat
12	Right	Profound, 94 dB	10	94	Profound
13	Bilateral	Profound, 97 dB	95	99	Profound
14	Bilateral	Severe, 83 dB	80	87	Profound
15	Right	Moderately severe, 62 dB	15	62	Flat
16	Bilateral	Moderate, 53 dB	55	51	Descending
17	Bilateral	Severe, 75 dB	79	71	Flat
18	Left	Severe, 78 dB	78	15	Flat
19	Bilateral	Severe Moderately severe, 62 dB	60	65	Decsending
20	Bilateral	Profound, 97 dB	95	100	Profound
21	Bilateral	Profound, 92 dB	95	90	Profound
22	Bilateral	Profound, 92 dB	94	90	Profound

Mean value of click-ABR threshold was 13 dB on left ears and 15 dB on right ears. Type A tympanometry was obtained 40 (90,9%) ears, while type C was obtained in the remaining four ears. Stapedius reflex was observed in 38 (86,4%) ears. An ENT examination and otoscopy showed no abnormalities.

**Figure 1.**
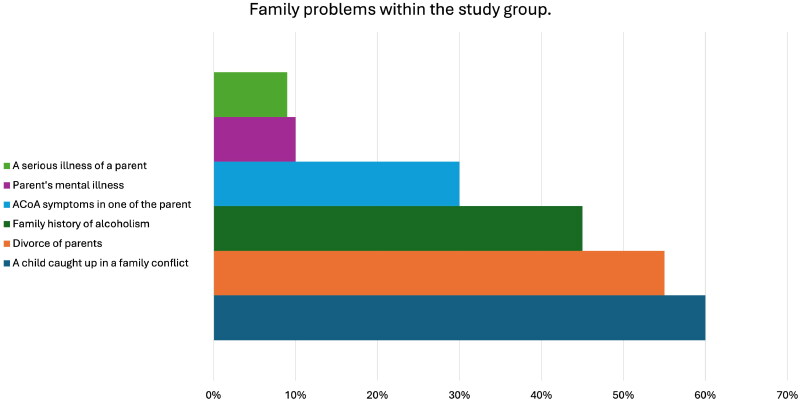
Family problems within the study group.

Alcoholism was present in 45% of families, a psychiatric disorder of one parent in 10% of families, life-threatening diseases of a parent – cancer, heart disease or obesity in 9% of families, divorce in progress in 55% of the families and conflicts in 60% of the families, adult children of alcoholics − 9%. The percentage distribution is shown in Figure 1.

**Figure 2.**
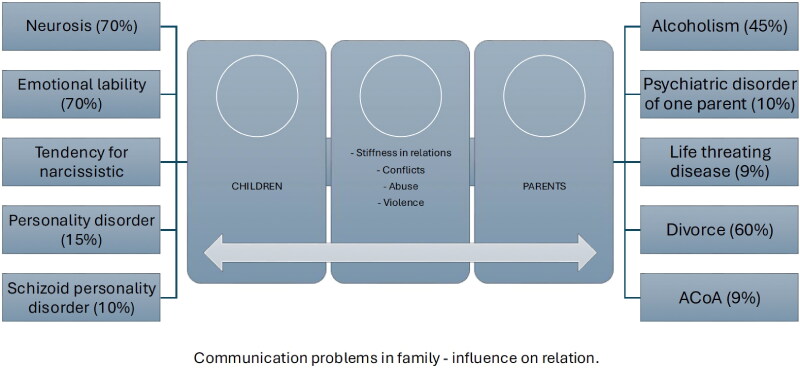
Communication problems in family influence on relation.

When we talk about adult children of alcoholics (ACoA), we mean people growing up in dysfunctional environments – families in which alcohol played a significant role, distorted the correct way of thinking and perception. People who grew up in a dysfunctional family model experience difficulties as adults and, it would seem, as independent people. The result of dealing with the alcohol addiction of a parent (or parents) is the formation of a phenomenon known as the ACoA. Alcoholism in the family deprives children of their own identity, self-worth and self-confidence. As adults, they continue to feel or behave like children, allowing others to violate their right to be independent and happy. The influence of communication is shown in Figure 2.

**Figure 3.**
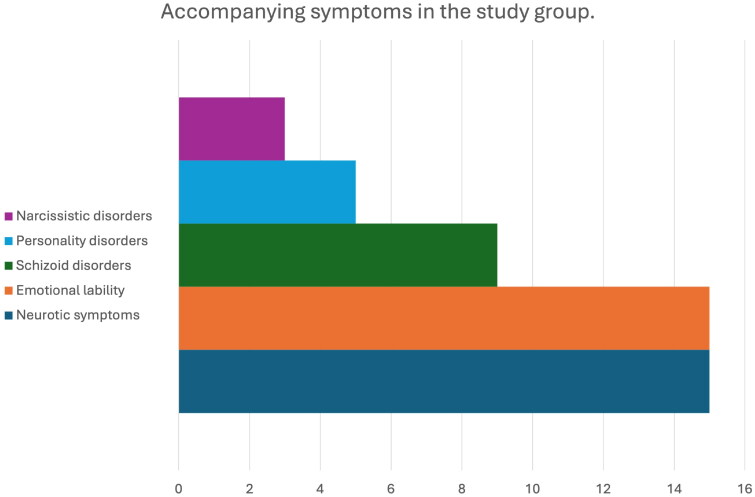
Accompanying symptoms in the study group.

In the examined group anxiety, psychasthenic and hypochondriac neurosis were present in 15 children, personality disorders - a tendency to emotional instability was present in 15 children, and a tendency for a narcissistic personality disorder was found in three children, traits of schizoid disorder were present in 9 children. Proportions of accompanying symptoms are presented in Figure 3.

Every child and his family in the examined group underwent talk therapy. 60% of families underwent family therapy and, in most cases, Erickson’s techniques of psychotherapy were applied [5]. The Ericksonian approach uses such non-specific techniques as hypnosis, the use of metaphor, provoking relapse, symptom work, symptom pre-scription, and many others. In addition, the patient should accept that he is a passive party and that most of the initiative lies with his therapist. In our patients, the symptoms waned during the hospital stay. Family and individual therapy were continued in a mental health outpatient clinic. Short-term psychotherapy and the Erickson approach were continued. A characteristic feature of the work of Ericksonian psychotherapists is the orientation to the resources and strength inherent in the patient and the possibility of using them on the way to a specific goal. During the sessions, the therapist often returns to earlier periods of life when the patient was functioning effectively, therapist also looks at how the patient coped with difficult life moments. Many people who come to therapy already have certain resources and strengths. Unfortunately, they use them only for the benefit of others or for various reasons, minimize their importance. Ericksonian psychotherapy is a kind of strategic work. Not only the patient but also the therapist initiates what happens during the meetings. The therapist identifies problems and actively encourages goal setting. He plans interventions to achieve them (often giving therapeutic tasks, for example). From time to time, he monitors the effects of the work.

Our therapy aimed to gain insight into the psychological causes of the disorder and unveil the emotions that remained repressed. Acquiring adequate response, strengthening family relations and stress management was also a goal. Recognizing and ap-prehending the rules of effective communication in the family. Recognizing methods of emotional expression. The effective psychological treatment techniques of psychogenic hearing loss have been established containing training for adequate reaction to symptoms that do not result from somatic disorder, attracting parents’ attention to the child’s emotions and ability to self-express and unblock emotions.

## Discussion

To exclude the anatomical background of psychogenic hearing loss several audiological tests should be performed, such as PTA, which is a subjective method, ABR, and OAE being the objective method. Cross-check method which was introduced by Jarger and Hayes stressed the importance of elaborated and various diagnostic methods and comparing their results to diagnose properly. Subjective auditory behaviour does not always reflect the actual hearing loss [12,13].

Also, in unilateral hearing loss magnetic resonance should be taken into consideration to exclude focal lesions of the brain – acoustic neuroma and others. In differential diagnosis, we must rule out auditory neuropathy, central processing disorder, and autism spectrum disorder, which leads us to a crucial psychological assessment [12,13].

Some authors differ on PHL inclusion criteria. According to Holenweg and Kompis one correct objective hearing test result and PTA hearing threshold increase enable the diagnosis of PHL [14,15]. We based on such criteria in our work.

Human physiology and psychology are complementary. Mind affects our body and body influences our mind. Every psychological process affects physiological reactions and internal organs functions. To recognize psychosomatic disorders in children and adolescents, it is important to realize that every emotion (anger, anxiety, happiness and sadness) reflects in behaviour, gestures, mimics, body posture and physiological function and organ processing. Accumulated energy that the child did not spend on physical activity may lead to organ malfunction and tissue damage [16]. Clinical somatic symptoms are associated with distress or impairment in everyday functioning. Somatization is often diagnosed as a somatic sign or somatic sign-related disorder affecting 8–12% of children and results in multiple negative functional consequences [17]. Functional disorders, which are a result of psychological or emotional factors, are called psychosomatic disorders. Somatization is a specialistic term that was introduced in psychology and medicine by psychoanalyst Wilhelm Steckl in the 1920s. In his understanding somatization stands for the ‘tendency to experience, conceptualize, and/or communicate psychological states or meanings as corporeal sensations, functional changes, or somatic metaphors’ [18].

The psychological approach towards the atypical causes of somatization has been recently renounced due to the generality and normalization of somatic sensations with an inclination to reference as ‘the sensation’ rather than ‘the symptom’ [15]. Walter Cannon was one of the pioneers of modern physiology and in particular a pioneer of physiological mechanisms of emotion [19].

His research showed that the human body is in permanent standby mode to react to unexpected perils. In case of emergency, the body reacts with the physiological adaptation mechanisms which are triggered by the function of the hypothalamus. The motoric, visceral and neurohormonal signals are simultaneously transferred to the cerebral cortex. During this reaction, a person can recognize emotions. Cannon stressed that prolonged standby mode and related anxiety may affect the efficiency of the body due to the possibility of developing functional or organic disorders in organs or systems [6]. It goes as far as to say that the common denominator of most psychosomatic disorders in children and adolescents is anxiety. Frightened children are reluctant to speak plainly about their fears but can manifest anxiety by their body complaining about heartache, tiredness, or constipation. Anxiety disorders constitute a group of various disorders which originate from the term ‘neurosis’.

According to the ICD-10 classification anxiety disorders are (F40) phobic anxiety disorders, (F41) other anxiety disorders, (F42) obsessive-compulsive disorder, (F43) reaction to severe stress and adjustment disorders, (F44) dissociative conversion disorders, (F45) somatoform disorders and (F48) other neurotic disorders. Functional hearing loss describes the malfunction of the hearing system without defining its cause. It is characterized by a disproportion between the subjective impression of hearing loss and the clinical state of a patient. The term ‘functional hearing loss’ is interchangeably used with psychogenic hearing loss and originates from a fragile mental state. Psychological diagnosis of psychogenic hearing loss refers to dissociative anaesthesia and sensory loss (F44.6) and somatoform disorders (F45) [4,20].

It turns out that psychogenic hearing loss affect usually children aged 7 to 16 and girls dominate in this group, 15 girls to seven boys, which is consistent with other authors [10,15–17,21,22]. Graf (1966) described the cases of children diagnosed with psychogenic hearing loss and defined characteristic circumstances which facilitate the manifestation of such symptoms:

1. A child is acquainted with another person who has hearing deficiencies or who has a history of transient hearing deficiency.
2. Parents or teachers suspect hearing loss.
3. Symptoms are accompanied by conflict at school or at home.
4. Intelligence of these patients is normal or above normal.
5. Symmetrical, flat hearing loss measured at around 40 to 80 dB.

In our study, the typical circumstances triggering psychogenic hearing loss were:

1. Alcoholism in the family – affected 45% of children.
2. Psychiatric disorder in one parent in around 10% of children.
3. Life-threatening diseases of the parent in around 10% of children.
4. Symptoms of Adult children of alcoholics in one parent in 30% of children.
5. Divorce in the past or divorce in progress in 55% of children.
6. Child involvement in the family conflict in 60% of children.

In the examined group, hearing deficit was observed at around 75–80 dB. Bilateral hearing impairment is more common than unilateral hearing impairment and affects up to 94% of patients [23]. Our study showed similar results – 40–80% of patients suffered from bilateral hearing loss. Typical psychological circumstances of psychogenic hearing loss can be defined by our observations. It is necessary to draw attention to these circumstances during the diagnostic process. The most common is hearing loss of the next of kin, problems at school or at home, death of a member of a family and separation of the parent. At least three of the aforementioned factors may suggest that the disorder we deal with is psychogenic hearing loss. In our study, 85% of children experienced conflicts in the family.

Yamamoto et al. (1990) examined 29 children suffering from psychogenic deafness from the audiological and psychological point of view. ABR examination turned out to be useful in the diagnostics process of such type of deafness. In our study, we obtained similar results because the ABR examination provided the background to exclude the anatomical background of hearing loss. Time that passed from the beginning of psychological therapy to recovery was significantly shorter than without this therapy − 7.5 of months compared to 17.1 months in the control group [23]. However, some studies show that 60% of children continue to experience increased hearing thresholds [3]. Psychological treatment revealed that the clinical course of psychogenic deafness in children appeared to be related to the patient’s personality and psychological stress.

Our research showed that functional disorders in children were accompanied by neurotic symptoms: anxiety, psychasthenic, hypochondriac neurosis – in 15 children, personality disorders: tendencies to emotional lability – in 15 children, tendencies to narcissistic disorders – in three children, tendencies to schizoid disorders – in nine children. Psychological studies showed that children with psychogenic deafness are usually related to an unconscious psychological defence mechanism as a reaction, for example, to school problems or family conflicts [3,24,25]. The goal of psychological therapy was to detect conflicting behaviours and treat them. These studies have shown that parental introversion combined with neuroticism is typical of the occurrence of psychogenic deafness.

Our study, as in Yamamoto et al. (1990), Aplin (1986), Beagley (1968), and Barr (1960), showed that family and personality problems cause unresolved difficulties in children and can lead to psychogenic deafness. Based on the observations from the studies, psychological treatment should be continued until the psychological conflict is resolved, even if the pure tone audiometry shows correct results. We have found that family therapy is very effective in the treatment of this type of disorder because the problems arise from the patient’s family situation. If psychological problems are not resolved other psychogenic disorders may appear, such as abdominal pain or headache.

We were able to establish diagnostic procedures and propose appropriate therapeutic procedures. First, the aim of this study was to exclude the anatomical basis of hearing loss in medical examinations and then to confirm the psychogenic basis of the hearing loss. The next step was to assess the scale of the child’s psychological problems, identify the conflict areas, and sources of frustrations and define the causes of pathological symptoms. The last stage was to adjust the appropriate psychotherapeutic treatment. The application of family and individual therapy proved to be very effective. In case of children who received individual psychotherapy, we did not observe any recurrence of the symptoms. On the other hand, in those patients whose parents did not cooperate during treatment, symptoms often lasted longer, and new psychosomatic symptoms appeared, such as headaches, heartaches, and pain in various parts of the body, most often in the ears. It turns out that the importance of family therapy in resolving a child’s emotional conflicts is very important. Family therapy is a form of assistance aimed at families, based on the main assumptions of the systemic approach. A family is perceived as a living psychosocial system in which every member interacts and influences each other. Thanks to those psychotherapeutic interactions parental competencies increase, bonds and relations between members of a family are rebuilt, mutual understanding and closeness are increased, and communication between members of the family improves. Members of the family become aware that they are able to overcome the difficulty and have the skills and resources to overcome difficulties in the future individually.

Therefore, the cooperation of the audiologist with mental health specialists is extremely important, as confirmed by other researchers [26–31].

This study has some unavoidable limitations. The main limitation is a small sample size with only 22 patients enrolled to the study. This may hinder the ability to detect significant results, impacting the study’s reliability and generalizability of results. However, the authors do believe that findings of the study offer new, potentially useful information about this rare condition.

## Conclusions

Psychogenic hearing loss is often neglected in the differential diagnosis of hearing disorders. In a difficult diagnostic process and treatment of psychogenic hearing loss disorder, the close cooperation of the audiologist, psychologist, patient, and his family is required. Ignorance of the psychogenic background of hearing loss often results in the preservation of incorrect behaviour patterns in children, delays start in targeted treatment and lead to economic deficits by running excessive diagnostic tests and administering unnecessary hearing aid fittings. Prolonged hospital stays, and excessive medical ex-aminations lead to unnecessary stress. It is crucial to identify psychogenic hearing loss in order to avoid overmedicalization and misdiagnosis. A diagnostic therapeutic pattern was established during a hospital stay.

**Figure 4.**
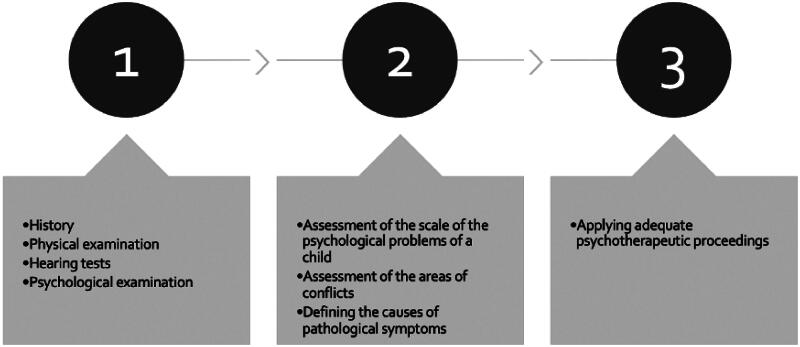
Diagnostic recommendation for patients with psychogenic hearing loss.

The diagnosis was based on an ENT examination, hearing tests, and psychological evaluation. The medical examinations aimed to exclude the anatomical background of hearing loss and therefore to confirm the psychogenic background of hearing loss. The next phase was to assess the psychological problems of the child, defining the source of conflicts and frustrations and defining the causes of pathological symptoms. The final phase was to apply adequate psychotherapeutic proceedings. Individual and family therapy proved to be the most efficient because the reasons behind somatization and somatization disorder are complex. Individual susceptibility to stress plays a major role. A child learns from their closest family and surrounding how to manage psychological stress and how to recognize and express emotions. If a child does not acquire such skills to a large extent, it may lead to a tendency to interpret physiological, bodily symptoms of experienced emotions as symptoms of a disease. Parental attitude towards a child is important. If a child is overly controlled, cannot live up to exaggerated expectations, or if parents focus overly on a somatic state of health, they can strengthen anxiety or stress which leads to consolidation of somatic symptoms. Recommendations for parents are shown in Figure 4. To avoid emotional problems including somatic disorders the ability to recognize and express emotions is essential. Acceptance of failures and motivation to seek a solution to problems instead of avoiding them, a good relationship with a child is a basic role of a parent. Constant interest demonstrated by a guardian not only when the child is causing troubles or is complaining of disease is the key to avoiding developing psychosomatic disorders such as psychogenic hearing loss.

## Data Availability

All results are available from the corresponding author.
